# Multimodal Surveillance Model for Enterovirus D68 Respiratory Disease and Acute Flaccid Myelitis among Children in Colorado, USA, 2022

**DOI:** 10.3201/eid3003.231223

**Published:** 2024-03

**Authors:** Kevin Messacar, Shannon Matzinger, Kevin Berg, Kirsten Weisbeck, Molly Butler, Nicholas Pysnack, Hai Nguyen-Tran, Emily Spence Davizon, Laura Bankers, Sarah A. Jung, Meghan Birkholz, Allison Wheeler, Samuel R. Dominguez

**Affiliations:** Children’s Hospital Colorado, Aurora, Colorado, USA (K. Messacar, M. Butler, H. Nguyen-Tran, S.A. Jung, M. Birkholz, S.R. Dominguez);; University of Colorado, Aurora (K. Messacar, H. Nguyen-Tran, S.R. Dominguez);; Colorado Department of Public Health and Environment, Denver, Colorado, USA (S. Matzinger, K. Berg, K. Weisbeck, N. Pysnack, E. Spence Davizon, L. Bankers, A. Wheeler)

**Keywords:** Enterovirus D68, EV-D68, surveillance, disease outbreak, viruses, Colorado, United States, acute flaccid myelitis

## Abstract

Surveillance for emerging pathogens is critical for developing early warning systems to guide preparedness efforts for future outbreaks of associated disease. To better define the epidemiology and burden of associated respiratory disease and acute flaccid myelitis (AFM), as well as to provide actionable data for public health interventions, we developed a multimodal surveillance program in Colorado, USA, for enterovirus D68 (EV-D68). Timely local, state, and national public health outreach was possible because prospective syndromic surveillance for AFM and asthma-like respiratory illness, prospective clinical laboratory surveillance for EV-D68 among children hospitalized with respiratory illness, and retrospective wastewater surveillance led to early detection of the 2022 outbreak of EV-D68 among Colorado children. The lessons learned from developing the individual layers of this multimodal surveillance program and how they complemented and informed the other layers of surveillance for EV-D68 and AFM could be applied to other emerging pathogens and their associated diseases.

Enterovirus D68 (EV-D68) causes epidemics of asthma-like respiratory disease and clusters of cases of the paralytic polio-like disease known as acute flaccid myelitis (AFM) (*1*). During summer/fall seasonal peaks, EV-D68 substantially strains healthcare resources with unexpected surges in emergency department (ED) visits, hospitalizations, and the need for intensive care unit (ICU)–level respiratory support for children (*2*,*3*). Detecting EV-D68–associated AFM cases relies on timely, targeted outreach to ensure prompt diagnosis, appropriate specimen collection and testing, and reporting to public health authorities (*4*). However, because of the inability of clinically available diagnostics to differentiate rhinoviruses from enteroviruses and the lack of widespread availability of EV-D68–specific testing, recognition of waves of EV-D68 infections is often delayed and the associated burden of disease remains substantially underdetected (*5*,*6*). Surveillance for EV-D68 is essential for early warning systems to guide responses to future waves of respiratory disease and AFM.

Although discovered in 1962 (*7*), EV-D68 was rarely detected before clusters of respiratory disease were reported in Europe, Asia, and the United States during 2008–2010 (*8*). In 2014, the largest and most widespread EV-D68 outbreak to date was reported in North America and Europe (*3*,*9*). During 2014–2018, a biennial pattern of circulation in the summer/fall was observed in the United States and Europe; the numbers of reported AFM cases increased with successive outbreaks (*10*–*12*). That biennial circulation pattern was disrupted during the COVID-19 pandemic; no substantial circulation was detected in the United States in 2020–2021, most likely because of the nonpharmaceutical interventions that were directed at curbing the spread of SARS CoV-2 (*13*). Modeling the growth of the population susceptible to EV-D68 during that period of limited activity suggested the potential for a larger outbreak when circulation returned (*14*).

After outbreaks in 2014 (*15*), 2016 (*16*), and 2018 (*17*), we established a multimodal surveillance program in Colorado for EV-D68 and AFM to better define their epidemiology and disease burden and to guide preparedness efforts (Figure 1). Our program included prospective syndromic surveillance for AFM and asthma-like respiratory disease, prospective EV-D68 clinical laboratory surveillance, and retrospective wastewater surveillance. The lessons learned from development and implementation of this multimodal surveillance system during the EV-D68 outbreak in 2022 (18) carry valuable implications for preparedness efforts for EV-D68 and other emerging pathogens.

**Figure 1:**
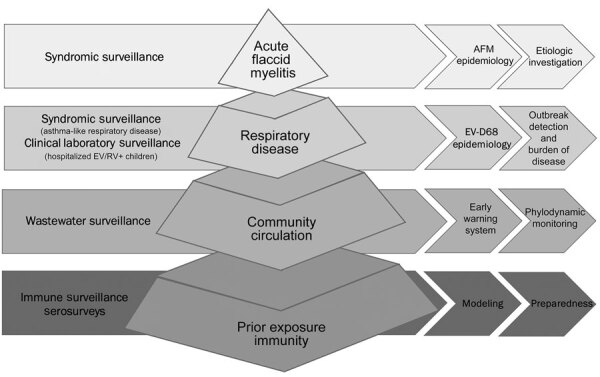
Multimodal surveillance model for enterovirus D68 in Colorado, USA. AFM, acute flaccid myelitis; EV, enterovirus; RV, rhinovirus.

## Methods

### AFM Syndromic Surveillance

AFM is a reportable condition statewide in Colorado as part of Centers for Disease Control and Prevention (CDC) nationwide AFM surveillance efforts (*4*). Healthcare providers are required to report suspected AFM cases to the Colorado State Department of Public Health and Environment (CDPHE) within 4 calendar days. Because there are no laboratory criteria for reporting AFM cases, syndromic criteria for reporting to public health authorities include any patient with new onset of focal limb weakness and magnetic resonance images (MRI) showing a spinal cord lesion with at least some gray matter involvement spanning >1 vertebral segments (*19*). CDPHE follows the Council of State and Territorial Epidemiologists guidance for AFM case ascertainment. Medical records and MRIs collected by CDPHE are ultimately classified by the CDC AFM neurology panel as confirmed, probable, or suspected in accordance with Council of State and Territorial Epidemiologists criteria.

### EV-D68 Respiratory Syndromic Surveillance

Beginning in 2018, we conducted ongoing near–real-time syndromic surveillance of asthma-like respiratory illness at Children’s Hospital Colorado (CHCO), a 444-bed quaternary care pediatric hospital in Aurora, Colorado; the hospital catchment area encompassed children in the Denver metropolitan area. Whereas upper and lower respiratory tract infection rates fluctuate with the circulation of many respiratory viruses, a surge in cases of asthma-like illness was specifically noted to coincide with the large EV-D68 outbreak in Colorado in 2014 (*2*); thus, medically attended asthma-like illness rates were subsequently tracked for syndromic surveillance of EV-D68 respiratory illness. A de-identified dataset of weekly ED visits with a principal billing diagnosis code of asthma (code J45.XXX from the International Classification of Diseases, 10th Revision, Clinical Modification) from CHCO was collected, and total weekly ED visits served as a denominator. We chose the diagnosis codes to reflect visits associated with an asthma exacerbation or asthma-like episode of wheezing that could be associated with EV-D68. To develop and determine the baseline for the forecast model, we obtained an identical retrospective dataset for the 3 years before the target surveillance year. From those retrospective data, we generated expected counts of weekly ED visits, indirectly standardized by age and sex. We then calculated a standardized morbidity rate for each week by dividing the observed asthma ED visits by the expected count. Our EV-D68 syndromic surveillance system consists of 2 components: a time series forecast model to predict the expected number of asthma ED visits each week and a cumulative sum chart procedure to serve as an alarm to identify potential temporal clusters of elevated weekly asthma ED visits (Appendix).

### Clinical Laboratory Surveillance

Clinical laboratory surveillance for EV-D68 respiratory illness was conducted during June–November 2022 among children at CHCO for whom residual respiratory specimens were positive for the enterovirus/rhinovirus target on the BioFire Respiratory Pathogen Panel 2.1 (BioFire Diagnostics, https://www.biofiredx.com). We selected specimens from hospitalized patients with enterovirus/rhinovirus respiratory disease and tested them by using an EV-D68–specific reverse transcription PCR (Appendix). We initially used a primer-probe set designed to target the 2014 B1 strain, in use at CHCO since 2015, for clinical surveillance testing during June–August of 2022. In August 2022, the PCR protocol was updated with primer and probe sequences designed to detect the predominantly circulating strain (subclade B3), as well as previously circulating strains, and used for all clinical laboratory surveillance testing (*20*).

### Wastewater Surveillance

We used the digital droplet PCR at the CDPHE laboratory to quantify EV-D68 virus concentration in wastewater samples collected twice weekly during June–December 2022. We used the updated PCR primer-probe set targeting EV-D68 subclade B3 noted above (Appendix). We included 3 sewersheds in the Denver metropolitan area in this analysis, referred to as utilities A, B, and C, which overlap with Adams, Arapahoe, Denver, and Jefferson Counties.

To examine the geospatial overlap in EV-D68 clinical laboratory case detections and detection of EV-D68 in wastewater, we linked residential postal (ZIP) codes of clinical cases to ZIP Code Tabulated Areas (ZCTAs) and conducted a descriptive analysis of the time and spatial relationship between positive clinical and wastewater detection of EV-D68. Our tabulation of positive EV-D68 clinical tests for the 3 select Denver metro area sewersheds where wastewater samples were collected (utilities A, B, and C) was based on the spatial overlap of the sewershed boundary and ZCTA boundary. To estimate allocation of cases to sewershed areas without exact address geolocation data, we split case counts among the sewershed areas (e.g., for a case from a ZCTA that overlapped 2 sewershed areas, we assigned a case value of 0.5 to each area). We used descriptive statistics to compare trends among the different layers of the surveillance system (Appendix).

## Results

**Figure 2:**
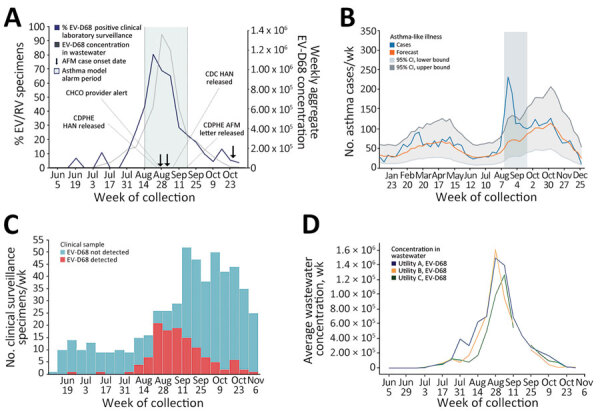
Multimodal surveillance during EV-D68 outbreak in Colorado, USA, 2022. A) Multimodal EV-D68 syndromic, clinical laboratory, and wastewater surveillance. To simplify the presentation of the temporal relationship between clinical positivity rates and the signal in wastewater, the viral concentrations of EV-D68 for the 3 utilities in this study are aggregated. B) Syndromic surveillance for asthma-like respiratory disease. C) Clinical laboratory surveillance for EV-D68 respiratory disease at CHCO. D) Wastewater surveillance for EV-D68 by wastewater utility service area. To generate the line presented in the graph, the concentration values for the 3 utilities were added together and averaged (mean) by sample collection date. The sample collection dates and cadence were uniform over time across all 3 utilities. The data are an estimation of the overall viral signal from the adjacent sewershed areas within the Denver metropolitan region (panel D; Appendix Figure 1). AFM, acute flaccid myelitis; CDC, Centers for Disease Control and Prevention; CHCO, Children’s Hospital Colorado; CHPHE, Colorado Department of Public Health and Environment; EV, enterovirus; HAN, Health Alert Network; RV, rhinovirus.

On August 14, 2022, the Colorado syndromic surveillance system for EV-D68 respiratory illness generated an alarm signal because the cumulative sum output exceeded the threshold for statistical significance during August 14–September 24, 2023 (Figure 2). That alarm signal coincided with an observed uptick in overall CHCO ED visits, hospital ward and ICU admissions, and enterovirus/rhinovirus detections from clinical testing of respiratory specimens. In August 2022, an updated primer-probe set designed against subclade B3 viruses detected EV-D68 in 5 respiratory specimens that the 2014 clade B1-targeted primer-probe set failed to detect (*20*,*21*). On the basis of those results, we converted all EV-D68 surveillance to the updated primer-probe set for all samples tested. Overall, EV-D68 detections were noted at low levels as early as June 19, 2022, increasing substantially the week of August 7, 2022, to a peak positivity rate of 78.6% in selected enterovirus/rhinovirus samples collected during the week of August 21, 2022. In total, 529 enterovirus/rhinovirus–positive clinical specimens were tested during June 15–November 3, 2022, and 121 (22.9%) were positive for EV-D68 (Figure 2).

After clinical laboratory surveillance confirmed EV-D68 as the cause of the enterovirus/rhinovirus spike in respiratory illness in Colorado, CHCO, CDPHE, and CDC coordinated local, state, and national public health responses. On September 1, 2022, CDPHE issued a statewide Colorado Health Alert Network message about EV-D68 circulation in Colorado, which contained education on AFM and reporting requirements. CHCO leadership activated a plan for emergency surge staffing and hospital bed availability for the expected increase in respiratory illness case volumes. On September 2, 2022, they released systemwide communications alerting providers of the EV-D68 outbreak and potential for AFM cases to follow. Early identification of the outbreak enabled advanced purchasing before the peak of the surge to help secure the CHCO supply chain for pediatric formulations of asthma medications and respiratory support supplies, which subsequently became a nationwide shortage. On September 9, 2022, after being alerted of increases in asthma-like illnesses and detection of sustained EV-D68 circulation by CDPHE and the CDC New Vaccine Surveillance Network sites, CDC released a nationwide Health Alert Network about severe respiratory illnesses associated with enterovirus/rhinovirus infections, including EV-D68 (*22*). In addition, a “Dear Provider” letter was mailed on September 20, 2022, to Colorado medical providers describing signs and symptoms of AFM and providing diagnosis and management recommendations and reporting requirements.

By late September 2022, EV-D68 respiratory syndromic surveillance showed decreasing levels of asthma-like illness cases in the CHCO ED, and clinical laboratory surveillance showed decreasing EV-D68 detection rates. The syndromic alarm signal de-activated, and observed rates returned to expected levels by mid-November in conjunction with EV-D68 clinical laboratory detections decreasing below 10%. The syndromic alarm period coincided with the increased EV-D68 circulation, and the alarm signal disappeared when the EV-D68 outbreak waned, even with a concurrent dramatic increase in CHCO ED visits and admissions resulting from a subsequent and overlapping, early, and large surge in respiratory syncytial virus bronchiolitis cases (Appendix Figure 2) (*23*).

Despite the substantial EV-D68 respiratory illness outbreak in Colorado and throughout the United States in 2022, the number of AFM cases was fewer than would be expected based on increases reported during previous years with substantial EV-D68 circulation. During 2022, CDC classified 4 suspected AFM cases that were reported in Colorado as confirmed or probable cases. In comparison, CDC confirmed 17 AFM cases in Colorado in 2018 and 11 in 2014 during peak years. Similarly, nationwide in the United States, 44 cases of AFM were confirmed in 24 states in 2022, compared with 238 in 42 states in 2018, 153 in 29 states in 2016, and 120 in 34 states in 2014, during peak years correlating with substantial EV-D68 circulation (*24*).

**Figure 3:**
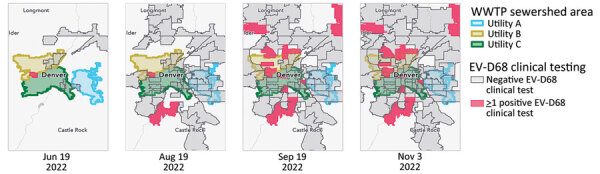
Temporal and geospatial correlation between clinical laboratory confirmed EV-D68 cases and wastewater detections, Colorado, USA, 2022. Cumulative positive EV-D68 clinical cases for June–November 2022 are shown by ZIP Code Tabulated Area overlaying Denver metropolitan area sewersheds. Data source: Children’s Hospital Colorado. EV, enterovirus; WWTP, wastewater treatment plant.

**Figure 4:**
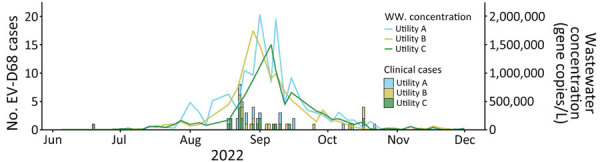
Temporal and geospatial correlation between clinical laboratory confirmed EV-D68 cases and wastewater detections, Colorado, USA, June–December 2022. Data source: Children’s Hospital Colorado. EV, enterovirus; WW, wastewater.

After the 2022 outbreak, CDPHE retrospectively tested wastewater for EV-D68 by using 117 samples from 3 facilities dating back to June 1, 2022, which were banked as part of the CDPHE Wastewater Surveillance Program. EV-D68 was first detected in wastewater on July 5, 2022, shortly after it was initially detected by clinical laboratory surveillance on June 19, 2022. Quantification and preliminary trend analysis of wastewater detection demonstrated an increasing trend in all 3 sampled sewersheds on July 18, 2022, nearly 1 month before the EV-D68 syndromic surveillance alarm was triggered. A similar temporal pattern followed the EV-D68 respiratory syndromic and clinical laboratory surveillance signals by 1–2 weeks (Figure 2) with geospatial and temporal correlation of ZCTA-level clinical laboratory EV-D68 case detections and detection of EV-D68 in wastewater from the corresponding sewersheds (Figures 3,4). 

## Discussion

We implemented a multimodal surveillance system in Colorado for EV-D68 and AFM, which promptly and accurately detected the large EV-D68 outbreak in the fall of 2022, enabling actionable, real-time surge planning and effective public health messaging. Each layer of surveillance independently provided unique insights into pathogen emergence, disease associations and burden, and community circulation; interdependently, the multiple layers of surveillance complemented each other with the potential to optimize performance and minimize limitations of the other layers in real-time in the future.

Rare, but severe, complications of emerging infectious diseases are often and appropriately the first to be recognized as public health priorities and therefore are typically the initial targets of surveillance to provide information about their epidemiology, etiology, and disease burden. The case definition for AFM was promptly constructed after the initial outbreak was reported in Colorado in 2014 (*25*). Subsequent national syndromic surveillance by CDC has been ongoing since that time; however, the reliance on astute clinicians to recognize, diagnose, and report suspected cases leads to continued case underascertainment (*19*). Substantial public health outreach efforts, including education campaigns (*26*), establishment of guidelines (*4*,*27*), and activation of local and state public health authorities and laboratories, have been used to improve recognition, reporting, and testing of AFM cases to support surveillance efforts. Through this pathogen-agnostic surveillance, EV-D68 was identified as the predominant pathogen driving the seasonal, biennial surges in AFM in the United States (*28*).

After a causal association was established (*29*,*30*), public health outreach efforts were focused on timely, targeted AFM education tied to periods of local EV-D68 circulation. Colorado enacted an enhanced AFM outreach program, which included local, state, and national notifications of EV-D68 circulation (*18*) and targeted provider outreach to heighten awareness of AFM during the 2022 outbreak. A large AFM spike was not detected in Colorado or the United States in 2022, which was the first time since 2014 that increased EV-D68 detection was not associated with increased AFM cases. Although much remains to be investigated with regard to the virologic, immunologic, and epidemiologic reasons behind that decoupling, the enhanced AFM surveillance enacted in Colorado was essential for establishing with confidence that the paucity of AFM reports during this period was most likely caused by a true lack of increased AFM cases in the community and not by a lack of recognition or failure to report.

In addition to syndromic surveillance for rare, severe complications, syndromic surveillance for more common presentations of an emerging pathogen can be used to signal outbreaks and improve knowledge of disease burden, especially for pathogens for which widespread testing is not available. During the 2014 outbreak, EV-D68–specific diagnostic testing was available only at CDC on enterovirus/rhinovirus–positive specimens from ICU-level patients with respiratory disease. Retrospectively, we established that syndromic surveillance of resource use for children with asthma-like respiratory diseases provided a better estimate of disease burden (*2*), which was used in an early warning system that sent an alarm as the first sign of an impending outbreak in 2022. Because of continued, limited, and selective sampling and testing for EV-D68, syndromic surveillance for asthma-like illness still provides the best estimate of EV-D68 disease burden. In 2022, that signal was also shown to specifically track with EV-D68, because it did not generate an alarm signal during the subsequent waves of respiratory syncytial virus, SARS-CoV-2, or influenza virus (Appendix Figure 2).

Clinical laboratory surveillance adds key insights into correlating syndromic signals with specific pathogens; however, it is reliant on test availability and performance. Although the enterovirus/rhinovirus signal from clinical testing is a useful early indicator, if this signal is used alone, EV-D68 epidemics can be misattributed to annual fall back-to-school rhinovirus resurgences. Detecting EV-D68 through clinical laboratory surveillance enabled early identification of the 2022 outbreak in Colorado compared with other centers in the United States, where difficulty interpreting the source of the enterovirus/rhinovirus spike on clinical platforms contributed to delayed recognition of the outbreak cause (J. Newland, PedsID ListServ, pers. comm, August 2022).

Until an EV-D68-specific target is included on commercial clinical testing platforms, the additional step of performing EV-D68–specific PCR on enterovirus/rhinovirus–positive specimens is necessary for clinical laboratory surveillance to confirm EV-D68 as the source of a respiratory disease outbreak as well as to detect lower level circulation that would not meet the alarm threshold of syndromic surveillance.

A key limitation of pathogen-specific PCR testing for newly emerging and constantly evolving RNA viruses is that primers must be matched to currently circulating strains to ensure adequate sensitivity. The lack of detection of the 2022 EV-D68 B3 strain by primers directed at the 2014 B1 strain demonstrated the value of the layers of syndromic surveillance in our multimodal system, because a syndromic signal that is not accompanied by PCR detection can alert clinical and public health laboratories to investigate, validate, and update primer-probe sets for detecting actively circulating strains. That iterative modification interdependently informed by our layered multimodal surveillance model enabled us to confirm that EV-D68 was the source of the 2022 respiratory outbreak and to assess the burden of disease among hospitalized children.

Wastewater surveillance and sequencing was initially developed for polio eradication, but scientific advancement has accelerated during the COVID-19 pandemic (*31*), serving as proof-of-principle of its public health utility for emerging pathogens, such as EV-D68 (*32*,*33*). Although our wastewater surveillance for EV-D68 was conducted retrospectively after the 2022 outbreak, we found direct temporal and geospatial correlation with our clinical laboratory surveillance from ZIP codes of hospitalized children with EV-D68 to validate this approach. Wastewater detections temporally preceded our syndromic surveillance alarm signal by 1–2 weeks, demonstrating future potential, if performed in real-time, to serve as the earliest warning of community circulation to detect an impending outbreak at the local level and could be expanded to track regional, national, or international spread.

EV-D68 is thought to be primarily transmitted and shed in respiratory secretions; fecal shedding is less common because most strains are acid-labile and degrade in the gastrointestinal tract (*7*). Our study is consistent with other published studies (*34*,*35*) that have demonstrated that even pathogens that are predominantly shed other than in feces, such as EV-D68, can still be detected and tracked through wastewater because of the high sensitivity of that method. Our study confirms that wastewater surveillance developed for poliovirus can be extended to EV-D68 and in the future probably beyond to other known and emerging enteroviruses associated with AFM. A key limitation to that approach is that enteroviruses, and many other pathogens, can be asymptomatically shed in feces and circulate among the community without causing substantial disease (*36*,*37*). That limitation can be overcome by the multimodal nature of our surveillance model by comparing detected strains in wastewater with those from clinical laboratory surveillance on specimens collected from patients with clinically relevant disease to verify that wastewater pathogen signals are of public health importance.

Last, a future component of multimodal surveillance being developed is the use of immunologic surveillance to assess the underlying immunologic background for an emerging pathogen. As a pathogen emerges, or reemerges, it is important to know the levels of prior exposure and immunity that may protect against future infection and affect transmission dynamics to inform epidemiologic models and predict future circulation patterns (*38*). Serosurveys to determine age-based seroprevalence can also help assess duration of maternal immunity, timing of primary exposure, and durability of humoral immunity (*39*), although they can be affected by antibody cross-reactivity (*40*). Those efforts are currently under way for EV-D68 through the PREMISE EV-D68 pilot study, which serves as proof-of-principle for an immunologic surveillance approach to pandemic preparedness to expedite preemptive development of countermeasures, such as monoclonal antibodies and vaccine candidates (*14*).

The multimodal surveillance system piloted for EV-D68 in Colorado is the culmination of several stepwise surveillance efforts implemented over the previous 8 years, which come with limitations. Differences in implementation timing, particularly the prospective versus retrospective nature, limit the ability to assess actionable effects of each layer. Differences in catchment between surveillance layers may influence correlation between signals. Our pilot surveillance program focused on 1 pathogen in 1 geographic region during 1 outbreak year; the model should be studied for other pathogens across broader regions in a prospective longitudinal manner to determine generalizability. Last, surveillance is meant to be actionable, but delay from signal detection to public health intervention diminishes potential effect and is a potential target for improvement.

Together, the layers of multimodal surveillance enacted in Colorado for EV-D68 rapidly detected the 2022 EV-D68 outbreak and enabled preparedness efforts for an effective local, state, and national response while creating the potential for more advanced future preparedness efforts. Actionable surveillance results enabled surge planning by hospital administration to increase staffing, hospital bed availability, and the supply chain for critical medications and also alerted providers to a potential influx of patients and provided recommendations to improve case recognition and clinical management. Although AFM cases were rare during the EV-D68 outbreak in 2022, our surveillance also demonstrates usefulness as an early warning system to trigger public health outreach efforts to enhance readiness to respond to future outbreaks of enteroviruses associated with AFM. Our multimodal approach, extending from surveillance for rare, severe complications to more common disease presentations and community circulation and immunity, demonstrates the value of investing in surveillance to inform preparedness to respond to the uncertainty that lies ahead with EV-D68 and other emerging pathogens.

Appendix Additional information for multimodal surveillance for enterovirus D68 outbreak among children, Colorado, USA, 2022.
