# Supplementary material for: Multimodal Surveillance Model for Enterovirus D68 Respiratory Disease and Acute Flaccid Myelitis among Children in Colorado, USA, 2022
Source: Emerg Infect Dis. 2024 Mar;30(3):423–31. doi: 10.3201/eid3003.231223 (PMC10902548; doi:10.3201/eid3003.231223)
Supplement: Appendix — Additional information for multimodal surveillance for enterovirus D68 outbreak among children, Colorado, USA, 2022. [file 23-1223-Techapp-s1.pdf]

Article DOI: https://doi.org/10.3201/eid3003.231223

EID cannot ensure accessibility for supplementary materials supplied by authors. Readers who have difficulty accessing supplementary content should contact the authors for assistance.

Multimodal Surveillance Model for Enterovirus D68 Respiratory Disease and Acute Flaccid Myelitis among Children, Colorado, USA, 2022

Appendix

Supplemental Methods

Syndromic Surveillance for EV-D68 Respiratory Disease

Our syndromic surveillance for EV-D68 respiratory disease consists of two components: a time series forecast model to predict the number of asthma ED visits each week, and a CUSUM chart procedure to serve as an alarm function to identify potential temporal clusters of elevated weekly asthma ED visits (*1–3*). The dependent variable in the forecast model is the square root of the weekly SMR, which transforms the outcome variable into an approximately normally-distributed random variable. We model this outcome variable during each week using a dynamic harmonic regression model with two Fourier components to account for seasonal trends and a first-order autoregressive model (ARIMA (1, 0, 0) errors) to model the smooth non-seasonal trend over time (*4*). The forecast model is estimated using the previous 3 years of data and out-of-sample predictions are made for the subsequent calendar year. The weekly forecast of asthma ED visits is obtained from the model by transforming the predicted outcome back to the SMR scale and multiplying the predicted SMR by the observed total number of ED visits for the target surveillance week. Within-sample residuals and out-of-sample forecast errors were evaluated for normality using the Shapiro-Wilks test, for constant variance over time using the Box-Ljung test, and for serial autocorrelation using plots of the autocorrelation function (ACF) and partial autocorrelation function (PACF). Dynamic harmonic regression is widely used in epidemiology and biostatistics to investigate seasonal patterns in disease occurrence (*4–7*).

We compare the observed weekly asthma cases to the model-based forecast by applying a Gaussian CUSUM statistic to the predicted outcome of the forecast model during the target surveillance week. The CUSUM statistic is a widely used surveillance technique for monitoring temporal processes (*3*). It is calculated by taking the cumulative sum of the standardized residuals of the forecast model and accounting for a downward drift parameter. The drift parameter can be viewed as the minimum change in the temporal process we are interested in detecting, and the value of the drift parameter is typically set to one-half the expected change or can be adjusted empirically (*3*,*8*). We calibrated the downward drift parameter by examining the standardized residuals of the forecast model during a previous asthma EV-D68 event in September 2014. During this event, the observed standardized residual was 6.68. Thus, we selected a value of 3.34 for the default drift parameter. However, since this event represented an unprecedented jump in asthma cases, we also monitor changes with the drift parameter set to 2.5 and 1.5, to serve as a medium and high sensitivity variant to the default settings. Values of the standardized residuals of the forecast model in excess of the drift parameter are cumulated by the CUSUM statistic. We designate our asthma surveillance model as statistically out of control if the CUSUM statistic is ≥3 standard residuals; however, in practice we monitor all CUSUM output in excess of 1 standard residual.

Clinical Laboratory Surveillance

EV-D68 surveillance testing on selected rhinovirus/enterovirus-positive specimens from 2015 through August of 2022 was performed as previously described (*9*,*10*). In August of 2022, surveillance testing was converted to an updated protocol using a primer-probe set designed to detect subclade B3 as well as previously circulating strains (*11*). Briefly, specimens selected for surveillance testing were spiked with Exogenous Internal Positive Control DNA (ThermoFisher, Waltham, MA, USA) and subjected to total nucleic acid extraction on the Qiagen EZ1 Advanced XL platform using the Virus 2.0 Mini Kit (Qiagen, Hilden, Germany). cDNA synthesis was performed using TaqMan Reverse Transcription Reagents with a $MgCl_2$ final reaction concentration of 4 mM (ThermoFisher, Waltham, MA, USA) on ABI Veriti Thermal Cycler (ThermoFisher, Waltham, MA, USA). PCR reaction components were as follows: qScript XLT One-step RT-qPCR ToughMix®, Low Rox (Quantabio, Beverly, MA, USA), primers and probes at a final reaction concentration of 250 nM, and internal positive control primers and probe (ThermoFisher, Waltham, MA, USA). Real-time PCR cycling conditions were 45°C x 10

minutes, 95°C x 10 minutes, followed by 45 cycles of: 95°C x 15 seconds and 60°C x 1 minute. A C_T value less-than 40 was considered positive for the EV-D68 target. A sample was considered negative if the EV-D68 target was not detected with a C_T value less than 40 and the internal positive control C_T value was less than 35. A sample was considered invalid if there was no amplification of the EV-D68 (Appendix Table 1).

Wastewater Surveillance

Extracted wastewater RNA for this study was provided by our collaborators at Colorado State University. The same EV-D68 quantitative PCR primer and probe design described above for clinical samples used to produce an amplicon of 94bps. Primer and probe concentrations and thermocycling conditions were modified for digital PCR on the Qiagen Qiacuity Eight® Instrument (Qiagen, Hilden, Germany) and analyzed using the instrument software. Briefly, primers and probes were used at a final reaction concentration of 500nM and 250 nM, respectively, and 10µl of extracted RNA was added per 40µl reaction using the Qiagen One-Step RT-PCR® Kit per manufacturers' guidance. De-identified clinical respiratory specimens previously confirmed to be positive for EV-D68 provided by Children's Hospital Colorado were used as a PCR positive control for this assay. A volume of 10µl of ddH_2O spiked into the PCR mastermix was used as a negative control for the PCR assay and only results where the NTC showed no amplification were considered. Digital PCR cycling conditions were 95°C x 10 minutes, 55°C x 5 minutes, followed by 45 cycles of: 95°C x 45 seconds and 55°C x 15 seconds, and a final 35°C for 1 min step.

Qiacuity instrument output is reported in copies/µl of the reaction volume. This output was converted to original sample concentrations by accounting for dilution of extract RNA in the PCR reaction, the concentration factors for Innovaprep concentration of 40mL of sample to ~400ul of eluate (100x) and RNA was then extracted using the Qiagen QiaAmp Kit paired with silica columns (Epoch Biosciences) eluted in ddH_2O (2.3x) and scaled to copies/L of original wastewater influent for reporting. The extracted total nucleic acid integrity and concentration of each processed sample was not assessed.

All wastewater samples were collected by a 24-hour composite autosampler and were retrospectively assessed for EV-D68 in singleton using minimal leftover extracted RNA or concentrate frozen from SARS-CoV-2 detection testing processing. Samples were spiked with

known quantities of bovine coronavirus (BCoV) as a recovery quality control metric. High level inhibition was absent from any samples tested, with 32.51+/−1.61% BCoV recovery across all three utilities during the study period. Limit of detection on the Qiacuity instrument for this assay was three positive partitions per well and limit of quantification was set at 4,000 copies/L of the EV-D68 target region based on absolute quantified copies/uL obtained using a 10-fold dilution series of de-identified Children's Hospital of Colorado confirmed EV-D68 clinical specimen spiked into ddH_2O run in triplicate with acceptable performance criteria defined as the lowest average absolute quantification at which the calculated relative standard deviation was <20%. *In silico* assessment of specificity was previously published (*11*) for this assay and was further assessed for this study using the NCBI primer BLAST and BLASTn online tool with no reasonable off-target detection concerns being identified.

Biobanked wastewater samples underwent Innovaprep concentration and were stored as eluate at −70°C for 3–9 months, depending on the sample collection date. All samples were thawed and treated uniformly and compared relative to each other by utility and sample collection date.

Given the lack of a standard viral concentration threshold to determine significant EV-D68 wastewater prevalence, a Bayesian Structural Time Series and Linear Model approach was employed to assess trends in EV-D68 concentration in wastewater over time to inform public health response. This approach was adapted from the current CDC National Wastewater Surveillance System guidance for assessing wastewater trends and parallels the approach used to determine trends in the viral concentration of SARS CoV-2 in Colorado. A trend category is determined based on the model fit for each sample. A 21-day window is used to determine the trend category for each sample; there must be at least five samples within this 21-day period to reliably determine the trend category for each individual sample. When the slope of the trend is greater than 0 and the p-value is <0.05, the trend is classified as *increasing*; when the slope is less than 0 and the p-value is <0.05, the trend is classified as *decreasing;* when the p-value is >0.05, the trend is classified as a *plateau.*

References

1. Romer Present PS, Berg K, Snow M, Richardson K. Detecting new sources of childhood environmental lead exposure using a statistical surveillance system, 2015-2019. Am J Public Health. 2022;112(S7):S715–22. PubMed https://doi.org/10.2105/AJPH.2022.307009

2. Sonesson C, Bock D. A review and discussion of prospective statistical surveillance in public health. J R Stat Soc Ser A Stat Soc. 2003;166:5–21. https://doi.org/10.1111/1467-985X.00256

3. Lawson AB, Kleinman K. Spatial and syndromic surveillance for public health. Chichester, West Sussex, England: John Wiley and Sons Ltd; 2005.

4. Hyndman RJ, Athanasopoulos G. Forecasting: principles and practice. 2nd edition, OTexts: Melbourne, Australia [cited 2022 Oct 20]. https://OTexts.com/fpp2

5. Wang L. Time series analysis and prediction of COVID-19 pandemic using dynamic harmonic regression models. Open J Stat. 2023;13:222–32. https://doi.org/10.4236/ojs.2023.132012

6. Ramanathan K, Thenmozhi M, George S, Anandan S, Veeraraghavan B, Naumova EN, et al. Assessing seasonality variation with harmonic regression: accommodations for sharp peaks. Int J Environ Res Public Health. 2020;17:1318. PubMed https://doi.org/10.3390/ijerph17041318

7. Chen R, Aschmann HE, Chen Y-H, Glymour MM, Bibbins-Domingo K, Stokes AC, et al. Racial and ethnic disparities in estimated excess mortality from external causes in the US, March to December 2020. JAMA Intern Med. 2022;182:776–8. PubMed https://doi.org/10.1001/jamainternmed.2022.1461

8. Dignam T, Hodge J, Chuke S, Mercado C, Ettinger AS, Flanders WD. Use of the CUSUM and Shewhart control chart methods to identify changes of public health significance using childhood blood lead surveillance data. Environ Epidemiol. 2020;4:e090. PubMed https://doi.org/10.1097/EE9.0000000000000090

9. Wylie TN, Wylie KM, Buller RS, Cannella M, Storch GA. Development and evaluation of an enterovirus D68 real-time reverse transcriptase PCR assay. J Clin Microbiol. 2015;53:2641–7. PubMed https://doi.org/10.1128/JCM.00923-15

10. Nguyen-Tran H, Reno S, Mwangi E, Mentel M, Hengartner R, Dominguez SR, et al. Qualitative detection of enterovirus D68 from PrimeStore® molecular transport medium: implications for home- and self-collection. Diagn Microbiol Infect Dis. 2023;106:115976. PubMed https://doi.org/10.1016/j.diagmicrobio.2023.115976

11. Ikuse T, Aizawa Y, Takihara H, Okuda S, Watanabe K, Saitoh A. Development of covel PCR assays for improved detection of enterovirus D68. J Clin Microbiol. 2021;59:e0115121. PubMed https://doi.org/10.1128/JCM.01151-21

Appendix Table 1. Enterovirus D68 (EV-D68) primer and probe sequences

Description	Sequence 5′ to 3′	Reference
EV-D68 Forward Primer	ACT GAA CCA GAR GAA GCY A	Ikuse 2021
EV-D68 Reverse Primer 1	AAA GCT GCT CTA CTG AGA A	Ikuse 2021
EV-D68 Reverse Primer 2	AAG GCT GCC CTG CTR AGA A	
EV-D68 Probe	/56-FAM/TC GCA CAG T/ZEN/N ATA AAY CAR CAY GG/3IABkFQ/	Ikuse 2021

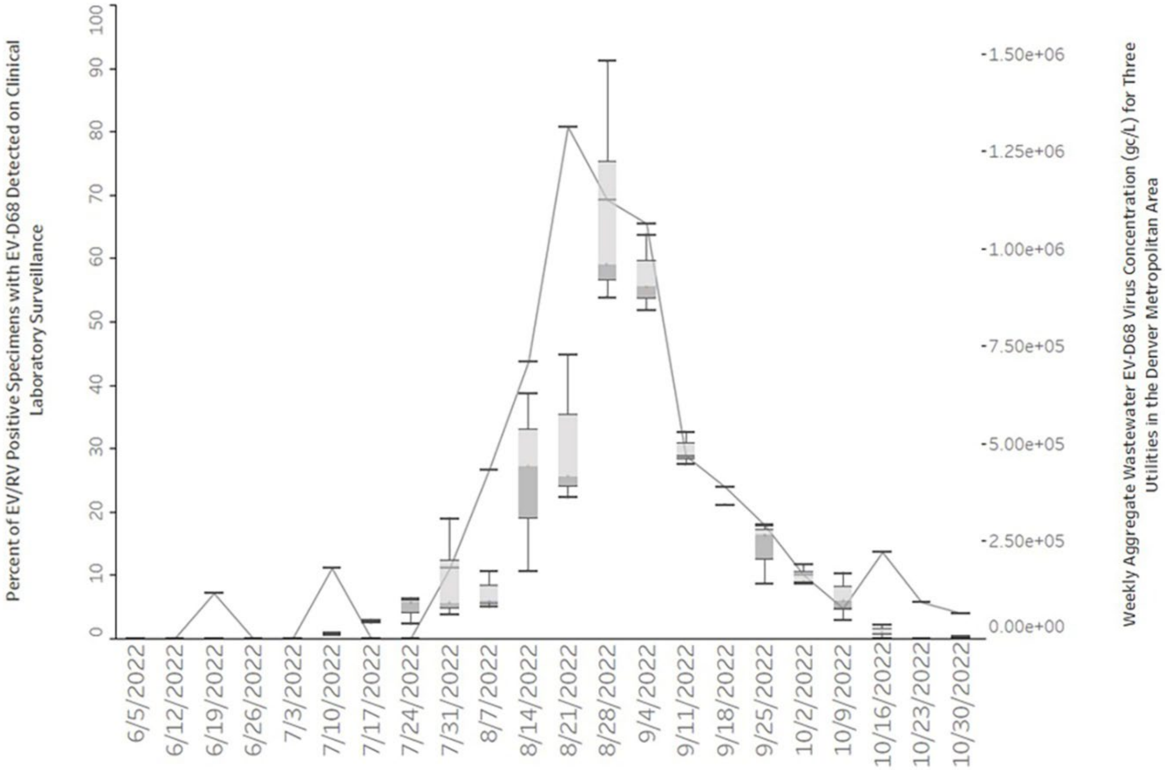

Appendix Figure 1. Detailed wastewater surveillance for EV-D68 from three wastewater utility service areas in the Denver metropolitan region and clinical laboratory surveillance for EV-D68 at Children’s Hospital Colorado. Clinical laboratory surveillance for EV-D68 among children with EV/RV respiratory disease at Children’s Hospital Colorado (solid line, L y-axis) and box plots for overall wastewater detections (R y-axis) from three utilities, sampled twice weekly, are displayed as data points per week of collection. This supplemental figure is used to display the individual data points and error bars to add additional detail to the data displayed in Figure 2A and 2D.

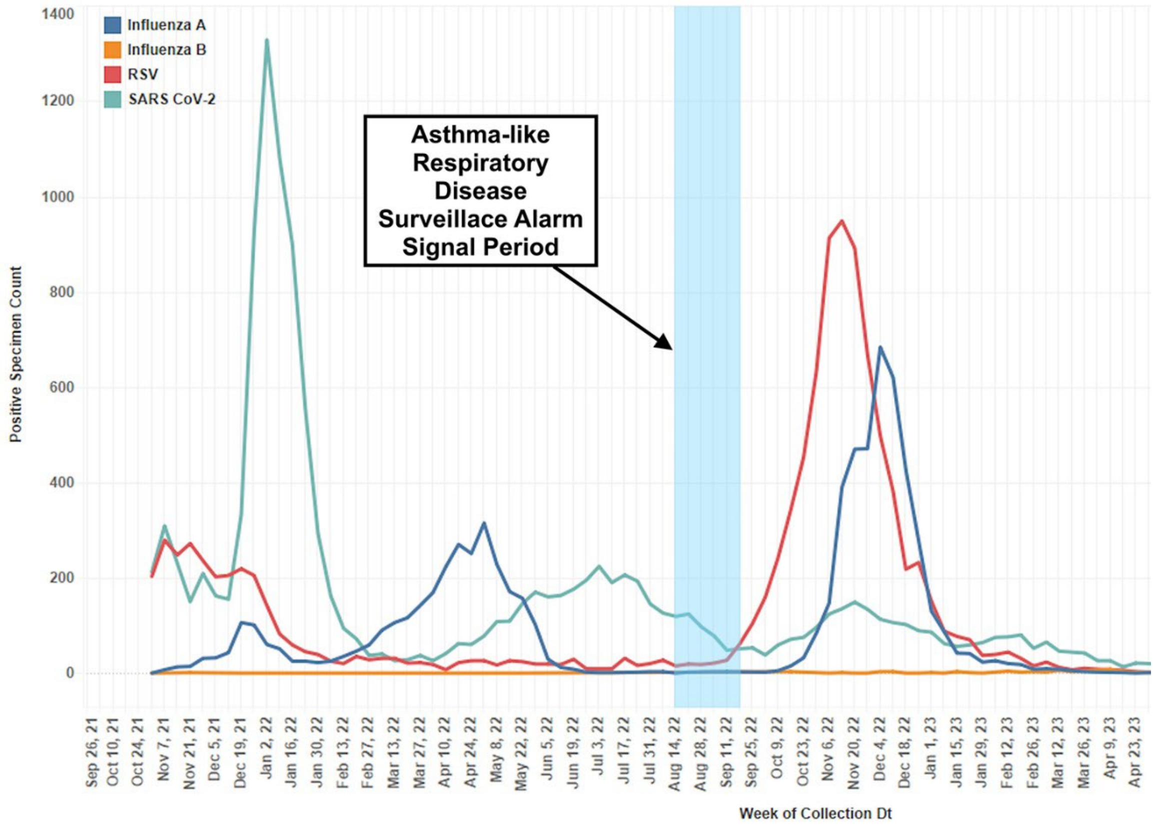

Appendix Figure 2. Clinical respiratory pathogen panel detections of influenza A and B, respiratory syncytial virus and SARS CoV-2 at Children's Hospital Colorado. Number of detections of influenza A, influenza B, respiratory syncytial virus and SARS-CoV-2 on respiratory pathogen panel testing at Childrens Hospital Colorado (colored lines) with alarm period from syndromic surveillance of asthma-like respiratory disease (shaded area).
